# Micromagnetic Study on Branch Hybridizations of Spin-Wave Modes in Ferromagnetic Nanostrips

**DOI:** 10.3390/ma15176144

**Published:** 2022-09-05

**Authors:** Binghui Yin, Mingming Yang, Xiaoyan Zeng, Ming Yan

**Affiliations:** 1 Department of Physics, Shanghai University, Shanghai 200444, China; 2 Department of Mathematics, Shanghai University, Shanghai 200444, China

**Keywords:** spin waves, magnetization dynamics, magnetic thin films, micromagnetic simulations

## Abstract

Magnonics is an emerging field in spintronics, aiming at the development of new-concept magnetic devices processing information via the manipulation of spin waves (SWs) in magnetic nanostructures. One of the most popular SW waveguides exploited currently is ferromagnetic nanostrips. Due to quantization caused by the lateral confinements, the dispersion of SWs propagating in a strip is characterized by a multi-branched structure. Consequently, SWs excited in the system involve superpositions of degenerate modes from different branches of the dispersion curves. In this work, we theoretically study the SW branch hybridization effect for two types of excitation methods, either by using a local oscillating magnetic field or a fast-moving field pulse. The former is based on the resonance effect and the latter on the Cherenkov-like emission mechanism. Micromagnetic simulations yield a variety of SW profiles with rather complex structures, which can be well explained by mode superpositions. These results draw attention to the significance of the SW branch hybridization effect when dealing with SWs in nanostrips and provide new aspects for the manipulation of SWs.

## 1. Introduction

Magnons are the quanta of spin waves (SWs), which are disturbances of magnetization that can propagate in magnetic systems. Magnonics, a branch of spintronics, aims to develop new-concept magnetic devices utilizing SWs to transmit or process information [1,2,3,4,5,6,7,8,9,10,11,12]. Due to the lack of Joule heat, magnonic devices would have the advantage of low power consumption compared to conventional electronic devices [8,9,10,11,12,13,14]. The core issue of magnonics is the manipulation of propagating SWs in particularly designed waveguides [10,11,12,15,16,17,18,19]. At this time, the most popular SW guide exploited is still long magnetic thin-film strips because of their simplicity for fabrication.

Similar to guided electromagnetic waves, SW modes traveling in nanostrips can form standing wave patterns in the transverse directions with quantized wave numbers. Since the strip is very thin, the thickness dependence can usually be ignored. However, the confinement along the width direction must be considered, which yields multiple branches of dispersion curves indexed by the corresponding order numbers of standing waves [10,20,21]. As a consequence, there is mode degeneracy in the system. In real experimental setups or numerical simulations, external stimuli must be applied to excite SWs traveling in the strip. Depending on the excitation method used, the output may contain multiple SW modes that are excited simultaneously. In this work, we systematically study the branch hybridization effect of SW modes when using two different excitation approaches, either by an oscillating field or a moving field pulse via the so-called spin-Cherenkov effect [22,23,24,25]. In the former case, the hybridization occurs because of the co-excitation of SW modes with different order numbers that are degenerate in frequency. In the latter case, a field pulse moving with a constant speed causes the co-emission of SW modes that are degenerate in phase velocity, resulting in branch hybridization in a different manner. We demonstrate the influence of the hybridization effect on the SW dispersion relations. Moreover, we show that the complex patterns of the hybrid modes obtained in our simulations can indeed be well reconstructed by superposing the relevant pure SW eigen modes.

## 2. Materials and Methods

We exploited a ferromagnetic thin-film strip, which was 10 nm thick, 100 nm wide and 2000 nm long, to serve as the SW waveguide. Note that the strip was long enough to allow the observation of SW propagations inside. Therefore, the modeled system with a finite length effectively represents an infinitely long wire. The magnetization dynamics of the system is governed by the Landau–Lifshitz–Gilbert (LLG) equation, given by

(1) $$\frac{d\vec{m}}{dt}=-\gamma \vec{m}\times {\vec{H}}_{eff}+\alpha \left[\vec{m}\times \frac{d\vec{m}}{dt}\right],$$

where $\vec{m}=\vec{M}/M_{s}$ is the normalized magnetization vector, γ is the gyromagnetic ratio, and ${\vec{H}}_{eff}\;$ is the effective field, including the exchange field, dipolar field, external field, and anisotropy field in principle. Micromagnetic simulations were performed by solving the LLG equation numerically to study the properties of SWs propagating in the strip. In simulations, the sample was discretized using a 1 nm cubic cell and the material parameters used were typical for Permalloy, with saturation magnetization $\mu_{0}M_{s}=1\;T$, exchange stiffness *A* = 1.3 × 10^−11^ J/m, damping factor *ɑ* = 0.01, and zero anisotropy. The simulations were performed using an open-sourced micromagnetic package MuMax3 [26,27]. In simulations, the time revolution of the magnetization field of the modeled system was calculated and recorded. By properly analyzing the obtained data, the properties of the dynamical processes of the magnetization, such as SW propagations, could be extracted and studied further.

**Figure 1:**
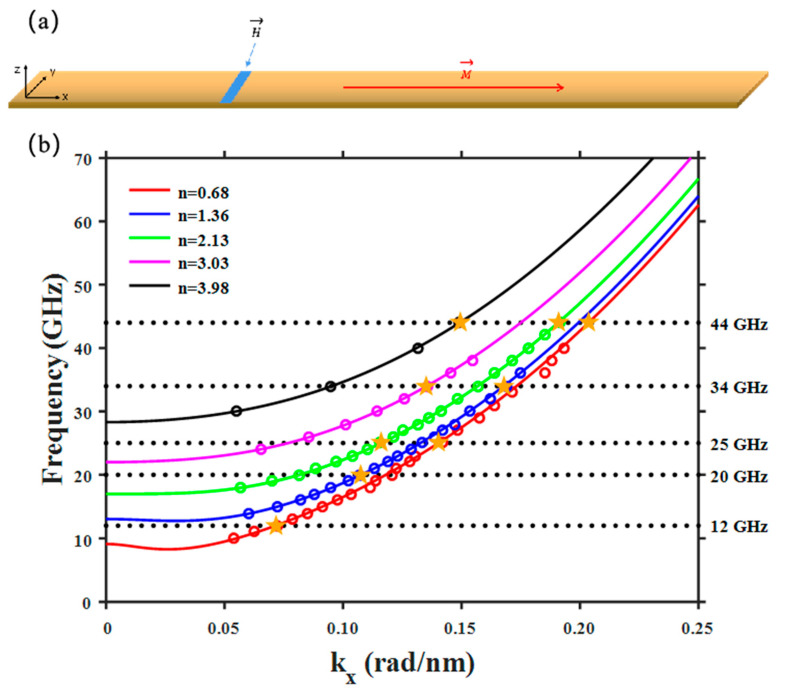
(**a**) A Permalloy thin-film strip magnetized along the positive *x* axis serving as an SW guide. An oscillating field is applied at the blue region to excite SWs. (**b**) Dispersion relations of SWs with different order numbers *n*. The dots are numerical data obtained in simulations while the solid lines are fittings using Equation (2). The dashed lines help to indicate the degeneracy of modes. The yellow stars indicate degenerate modes that in principle can be co-excited by a particular oscillating field.

Due to the shape anisotropy, the magnetic strip in equilibrium was magnetized along the longitudinal direction (*x* direction) as shown in Figure 1a. We used two methods to stimulate SWs in the system. The first one involved applying a localized magnetic field oscillating with a particular frequency, which can excite monochromatic SW modes. The other one involved applying a localized field pulse moving with a uniform velocity, which can excite SW modes propagating with the same phase velocity [22,23]. In the next section, we discuss our simulation results of these two excitation methods separately, focusing on the branch hybridization effect of SW modes.

## 3. Results

Before showing our simulation results, we will first explain the dispersion curves of SW modes traveling in a thin-film strip and its influence on the excitation of SWs. Due to the lateral confinements in the transversal direction (*y* axis in Figure 1a), the corresponding wave vector is quantized, given by $k_{y}=n\pi /w$, where *w* is the width of the strip and *n* a positive integer, defining the order number of the standing wave formed in the transversal direction [28]. For each *n*, there is a dispersion curve with respect to the continuous wave vector $k_{x}$ in the longitudinal direction of the strip (*x* axis in Figure 1a), thus yielding a multi-branched structure (can be seen in Figure 1b). We point out that a quantization in the thickness direction (*z* axis in Figure 1a), which would result in much higher frequency, is ignored in our consideration.

In simulations or experiments, one may excite a monochromatic SW mode by applying an oscillating field with a particular frequency. However, the frequency degeneracy of SW modes with different order numbers *n* leads to multiple excitations simultaneously, resulting in branch hybridizations. In our simulations, we applied local fields with specially designed spatial distributions to maximally couple to a mode with a particular order number [29]. For instance, a field uniformly distributed across the strip is supposed to excite a *n* = 1 mode. By varying the frequency and shape of the oscillating fields, the dispersion curves with respect to $k_{x}$ can be calculated numerically. The results obtained are drawn in Figure 1b (dots), showing five dispersion curves in total (*n* = 1~5).

In fact, the dispersion relation of SW modes in a longitudinally magnetized stripe can be calculated analytically neglecting the quantization along the thickness direction [20,30,31,32,33], with the formula given by

(2) $$\omega =\gamma \sqrt{\left(A^{*}M_{s}k^{2}+M_{s}p\sin^{2}\varphi \right)\left(A^{*}M_{s}k^{2}+M_{s}\left(1-p\right)\right)},$$

where γ is the gyromagnetic ratio, $A^{*}=2A/\mu_{0}M_{s}^{2}$, the wave numbers $k^{2}=k_{x}^{2}+k_{y}^{2}$, $p=1-\frac{1-e^{-kd}}{kd}$ where *d* is the thickness of the wire, and $\sin^{2}\varphi =k_{y}^{2}/k^{2}$. Note that $k_{y}=n\pi /w$, which is quantized and yields multiple branches of the dispersion. As indicated by the dashed lines in Figure 1b, except for frequencies below a certain value, there are always multiple modes with different order numbers that are degenerate in frequency.

The dots in Figure 1b are numerical data obtained in simulations, while the solid lines are fittings using Equation (2). As we will show later, the SWs excited in simulations are not pure eigen modes with a definite order number, but actually the superposition of several degenerate modes with different order numbers. As a consequence, there is a clear discrepancy between simulations and analytical calculations in terms of the dispersion relations. Note that, to fit the numerical results, the order numbers *n* used in fitting are deviated from integer numbers as shown in Figure 1b.

**Figure 2:**
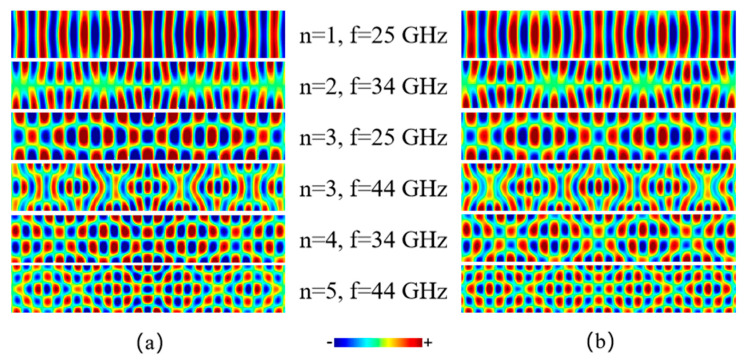
(**a**) SW patterns extracted from simulations when excited by using local fields oscillating with a particular frequency *f*. Each local field has a specially designed spatial distribution to couple maximally to a mode with a certain order number as indicated by *n*. These patterns are clearly more complex than regular standing waves along the width direction. (**b**) Wave patterns reconstructed artificially by superposing several degenerate eigen modes with different order numbers, demonstrating the branch hybridization effect taking place in simulations.

We now demonstrate the branch hybridizations observed in our simulations. Although we tried to excite SW modes with a definite order number from 1 to 5 using local fields with different spatial distributions, the actual wave patterns obtained are clearly more complex than those with a simple standing wave pattern along the width direction, as shown in Figure 2a. The spatial configurations of the SW modes shown here and thereafter are snapshots of the out-of-plane component (*z* component) of the magnetization, which are taken when the modes are fully developed in the system after excitation. We argue that these wave patterns are resulted from the superposition of degenerate modes that are excited simultaneously by the oscillating field applied in simulations. For instance, when applying a 44 GHz field as shown in Figure 1b, there are five degenerate modes in total (*n* = 1~5) that in principle can be excited all together. The amplitude of each excited mode depends on the strength of the coupling between the mode and the exciting field. The actual wave patterns obtained in simulations are therefore the superposition of various modes with different amplitudes.

In fact, the wave patterns obtained in our simulations can be artificially reproduced as shown in Figure 2b. This was done by superposing several pure eigen modes with different integer order numbers mathematically. For example, the wave pattern for *n* = 5, *f* = 44 GHz was reconstructed by using two pure eigen modes (*n* = 3, 5) with degenerate frequency, as indicated by the yellow stars in Figure 1b. Note that other degenerate modes with even order numbers are not relevant here because the spatial distribution of the excitation field applied is symmetric with respect to the central line of the strip, which therefore does not couple to SW modes that are antisymmetric. Although the excitation field in this case is designed to maximally couple to the *n* = 5 mode, it must also couple to other modes with odd order numbers. As shown in Figure 2b, the wave pattern for *n* = 5, *f* = 44 GHz is almost perfectly reproduced by superposition using a ratio of 7:3 between the amplitude of the pure *n* = 5 mode and the *n* = 3 one. For comparison, the wave pattern (*n* = 3, *f* = 44 GHz) which is excited by applying a field designed to maximally couple to the *n* = 3 mode can also be well reproduced by adjusting the ratio between the amplitude of the *n* = 5 mode and the *n* = 3 one to 3:7. In a similar way, other wave patterns shown in Figure 2a can be reproduced as well. This clearly demonstrates that the excited SWs that propagate in the strip are not pure eigen modes with definite order numbers, but hybrid ones resulting from the co-excitation of degenerate modes.

**Figure 3:**
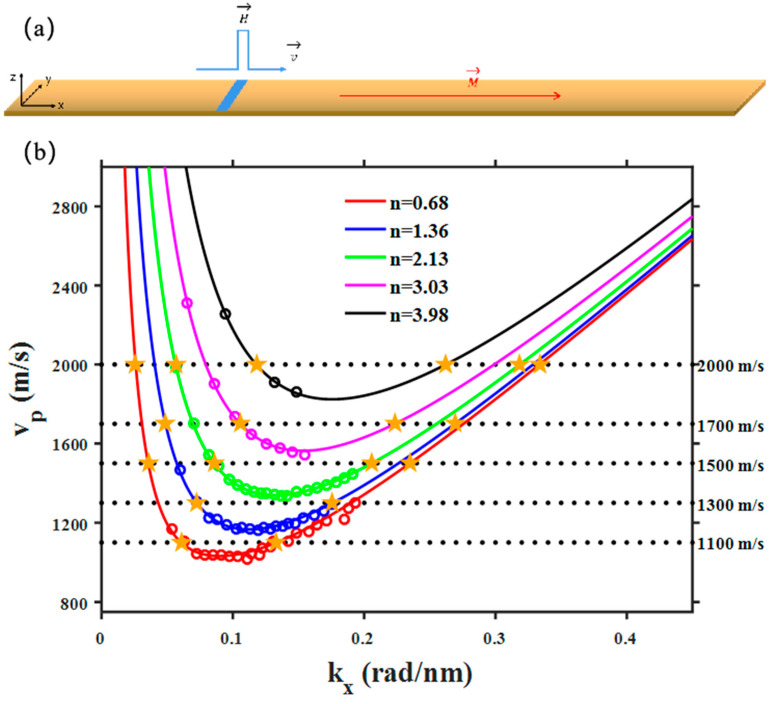
(**a**) A localized field pulse (blue) moving along the strip to generate Cherenkov-like excitation of SWs. (**b**) SW phase velocity versus wave vector extracted from the dispersion relation. Dots are numerical results and solid lines are fittings using Equation (2). Dashed lines help to identify multiple degeneracy of phase velocity of SW modes with different order numbers *n*. The yellow stars indicate modes that are degenerate in phase velocity that in principle can be co-excited by a particular moving field pulse.

We further study the branch hybridization effect with an alternative excitation mechanism, the SW Cherenkov effect [22,23]. A perturbation to the magnetic system moving with a speed exceeding the minimum SW phase velocity would cause strong SW excitations, similar to the Cherenkov radiation of lights or the sonic boom. As shown in Figure 3a, a moving field pulse can easily generate Cherenkov-like excitation of SWs. This type of excitation is characterized by the exact match between the SW phase velocity and the speed of the moving pulse. From the SW dispersion, one can easily extract the SW phase velocity dependence of the wave vector, as shown in Figure 3b. Again, the dots are numerical results and the lines are analytical fittings using Equation (2). For each SW branch (with a definite *n*), there are two modes that are degenerate in velocity, which can both be excited by a moving field pulse traveling with the same speed. Due to their different group velocity, the mode with a large (small) wave vector will move in front of (behind) the pulse [22,23].

**Figure 4:**
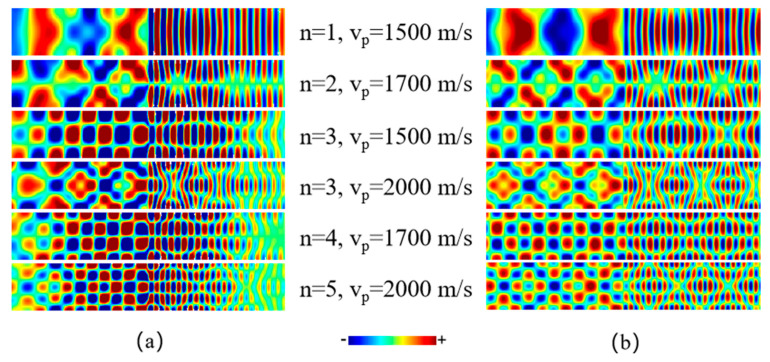
(**a**) SW patterns obtained in simulations which are excited by moving field pulses with different speeds. Each field pulse has a specially designed spatial distribution to couple maximally to a mode with a certain order number as indicated by *n*. (**b**) Wave patterns reproduced by artificially superposing several pure eigen modes with same phase velocity and different order numbers.

It can be seen from Figure 3b that if the pulse speed is large enough, the branch hybridization effect should also take place because of the multiple degeneracy of SW phase velocity. For instance, SWs excited by a pulse moving with a speed of 2000 m/s will in principle involve the superposition of at least five modes both in front of and behind the pulse. Again, this is clearly illustrated in Figure 4a, which shows complicated wave patterns obtained in simulations when excited by using different pulse speeds. Similar to the case of using an oscillating excitation field, these wave patterns obtained in simulations can also be well reproduced by artificially superposing relevant SW modes with the same phase velocity yet different order numbers, as shown in Figure 4b.

## 4. Discussion

Finally, we point out a unique feature of the SW branch hybridization in the Cherenkov-type excitation case. When considering the interference of two waves with different frequencies in a common situation, the resulting wave acquires a so-called modulation speed that is different from either of the phase velocities of the two waves. However, in our case, although all the modes involved in the hybridization have different frequencies and wave vectors, they share exactly the same phase velocity due to the merit of the excitation mechanism. Consequently, the modulated wave has a modulation speed that is exactly equal to the phase velocity of all the modes. This can be easily proven as

(3) $$v_{h}=v_{p1}=v_{p2}=v_{p}=v_{m},$$

where $v_{h}$ is the pulse velocity, $v_{p1}$ and $v_{p2}$ are the phase velocities of two monochromatic SWs, with $v_{p1}=\omega_{1}/k_{x1}$ and $v_{p2}=\omega_{2}/k_{x2}$, $v_{p}=\left(\omega_{1}+\omega_{2}\right)/\left(k_{x1}+k_{x2}\right)$ is the phase velocity of the modulation wave, and $v_{m}=\left(\omega_{1}-\omega_{2}\right)/\left(k_{x1}-k_{x2}\right)$ is the modulation velocity of the modulation wave.

**Figure 5:**
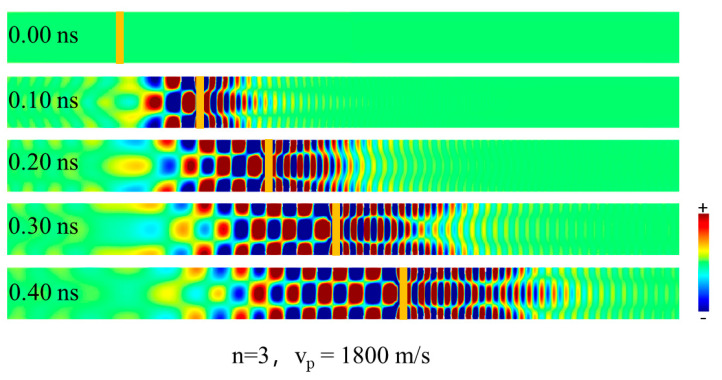
Time-serial snapshots of SW pattern excited by a field pulse moving with a speed of 1800 m/s. The yellow bar indicates the location of the moving field pulse at different times.

Figure 5 show several snapshots of the SW excitation using an 1800 m/s moving pulse. Clearly, the wave patterns both in front of and behind of the pulse move precisely together with the pulse, indicating the equivalence between the modulation speed and phase velocity.

## 5. Conclusions

In summary, we studied the branch hybridization effect of SWs propagating in a commonly used wave guide, i.e., a magnetic thin-film strip, by using micromagnetic simulations. To a certain extent, such an effect is unavoidable due to the lateral confinement of the magnetic sample resulting in a multi-branch structure of the SW dispersion relation. Our results show that the branch hybridization can lead to modification of the SW dispersion relation and more significantly the spatial configurations of SWs traveling in the strip either excited by using an oscillating field or a moving field pulse. As a matter of fact, the complicated wave patterns obtained in our simulations can be almost perfectly reproduced by the superposition of different eigen modes that are degenerate either in frequency or phase velocity, which unambiguously proves the branch hybridization effect in magnetic thin-film strips. From an application point of view, special attention must be paid to the branch hybridization effect in the development of novel spintronic devices utilizing ferromagnetic strips as SW guides. On the other hand, this effect also provides a new approach for the manipulation of SWs in nanostructures.

## Data Availability

Not applicable.
